# Insights into the quality of recombinant proteins produced by two different *Bombyx mori* expression systems

**DOI:** 10.1038/s41598-022-22565-7

**Published:** 2022-11-02

**Authors:** Hiroyuki Kajiura, Ken-ichiro Tatematsu, Tsuyoshi Nomura, Mitsuhiro Miyazawa, Akihiro Usami, Toshiki Tamura, Hideki Sezutsu, Kazuhito Fujiyama

**Affiliations:** 1 International Center for Biotechnology, Osaka University, 2-1 Yamada-Oka, Suita-Shi, Osaka, 565-0871 Japan; 2 Institute for Open and Transdisciplinary Research Initiatives (OTRI), Osaka University, 2-1 Yamada-Oka, Suita-Shi, Osaka, 565-0871 Japan; 3 Division of Silk-Producing Insect Biotechnology, Institute of Agrobiological Sciences, National Agriculture and Food Research Organization, 1-2 Owashi, Tsukuba, Ibaraki 305-8634 Japan; 4 Sysmex Corporation, 1548 Ooaza Shimookudomi, Sayama, Saitama 350-1332 Japan; 5 Division of Biomaterial Sciences, Institute of Agrobiological Sciences, National Agriculture and Food Research Organization, 1-2 Owashi, Tsukuba, Ibaraki 305-8634 Japan; 6 Silk Science and Technology Research Institute, 1053, Iikura, Ami-Machi, Ibaraki, 300-0324 Japan; 7 Osaka University Cooperative Research Station in Southeast Asia (OU:CRS), Faculty of Science, Mahidol University, Bangkok, Thailand

**Keywords:** Glycobiology, Expression systems

## Abstract

The silkworm, *Bombyx mori*, is an attractive host for recombinant protein production due to its high expression efficiency, quality, and quantity. Two expression systems have been widely used for recombinant protein production in *B. mori*: baculovirus/silkworm expression system and transgenic silkworm expression system. Both expression systems enable high protein production, but the qualities of the resulting recombinant proteins have not been well evaluated. In this study, we expressed bovine interferon γ (IFN-γ) using the two systems and examined the quality of the resulting proteins in terms of *N*-glycosylation and protein cleavage. Both expression systems successfully produced IFN-γ as an *N*-glycoprotein. Although the production in the baculovirus/silkworm expression system was much more efficient than that in the transgenic silkworm expression system, unexpected variants of IFN-γ were also produced in the former system due to the different *N*-glycosylation and C-terminal truncations. These results indicate that while high protein production could be achieved in the baculovirus/silkworm expression system, unintentional protein modification might occur, and therefore protein expression in the transgenic silkworm expression system is preferable from the point-of-view of *N*-glycosylation of the recombinant protein and evasion of unexpected attack by a protease in *B. mori*.

## Introduction

In response to the increasing demands for protein therapeutics for human use, many biopharmaceutical protein productions have been attempted using various production hosts, and many of the resulting products have been approved^1^. These biopharmaceutical protein productions have traditionally relied on bacterial fermentation or mammalian cultured systems, but they have been hampered by various host-specific limitations with respect to scalability, production costs, contamination risk, and post-translational modifications^2,3^. In fact, in regard to contamination risk, it has been reported that an accidental virus infection of mammalian cells in a manufacturing facility spoiled a bioproduction run of therapeutic proteins and ultimately resulted in improper treatment of patients^4^. Therefore, there is an urgent need for improved alternative protein production platforms.

The establishment of various cell lines, mainly lepidopteran cell lines, and protein expression systems utilizing baculovirus that encode a gene of interest have enabled the use of insect cells as protein production hosts in basic research as well as in the manufacture of biologicals for human and veterinary use^5,6^. However, the use of insect cells for protein production still has disadvantages in terms of maintenance and production costs, the complexity of media, potential protein degradation caused by proteases, and the risk of mammalian virus infections^3,7^. Moreover, the identification of contaminant viruses in insect cells^8–11^ has rendered these cells less desirable as a biopharmaceutical protein production system for human use, although two of these viruses identified flock house virus and Sf-rhabdovirus are not considered to be mammalian pathogens^5^. In contrast, the silkworm *Bombyx mori* was recently identified as an attractive insect host with promise for recombinant proteins production. Although the *B. mori* latent virus (BmLV) was found in *B. mori*-cultured BmN cells^12^, and three rhabdovirus-like sequences on *B. mori* were identified^6^, thus far, neither BmLV proliferation nor the presence of the three rhabdovirus-like viruses in larvae has been reported. These facts suggest that *B. mori* larvae are free from virus infection, and that recombinant protein production in *B. mori* larvae meets the biosafety standards required for biopharmaceutical production. Owing to the establishment of two silkworm expression systems a baculovirus expression system using *B. mori* nucleopolyhedrovirus and silkworm larvae^13,14^ and a transgenic gene-expression approach using microinjection of a gene of interest into eggs in conjunction with a transposon *piggyBac*-based system^15^, the tremendous capabilities of silk production can now be applied to recombinant protein production. Using these methods, *B. mori*-produced interferon (IFN) was introduced as the first antiviral drug for veterinary use, and two *B. mori*-produced IFNs are currently on the market^16^. Protein production in *B. mori* enables breeding at high density in a closed system, higher expression efficiency than that by bacterial expression, less production differences between batches, and protein post-translational modifications similar to those in mammals. In addition, the use of a GAL4/UAS system in combination with a *piggyBac* system has enabled spatiotemporal-specific expression of transgenes of interest^17^, which also facilitates easy purification of the recombinant protein. Since the development of these techniques, various kinds of recombinant proteins have been expressed using organ-specific protein production^18,19^.

In the case of protein production in *B. mori*, the biological activities of the recombinant proteins should be reevaluated. In *B. mori*, two expression systems, a baculovirus-based expression system using silkworm as an expression host (baculovirus/silkworm expression system) and a transposon-based transgenic gene expression system (transgenic silkworm expression system), are widely used, and the proteins produced by these systems might have different properties. Both expression systems enable high protein production, but their respective effects on the recombinant protein qualities, especially post-translational modifications such as protein degradation, have not been examined. In general, the baculovirus/silkworm expression system yields much greater amounts of protein than the transgenic silkworm expression system, but this augmented production might provoke endoplasmic reticulum (ER) stress and/or dysfunction via unfolded protein accumulation, which in turn could trigger ER-associated degradation and/or an unfolded protein response to restore ER homeostasis^20,21^. Indeed, co-expression of molecular chaperones with a target gene of interest has been shown to facilitate and enhance recombinant protein production in *B. mori*^22,23^, suggesting that the endogenous quality-control machinery of the ER in *B. mori* is not sufficient for the production of correctly folded proteins. Furthermore, the introduction of a baculovirus into the baculovirus/silkworm expression system itself could severely damage *B. mori* and induce cell death. If any of these potential hazards were to arise, the baculovirus/silkworm expression system could produce low quality, post-translationally unprocessed, immature or proteolytically-degraded proteins.

In this study, to focus on the quality of the recombinant proteins produced in *B. mori* by the two systems, we expressed bovine interferon γ (IFN-γ) as a model protein in the baculovirus/silkworm expression system and transgenic silkworm expression system. The IFN-γ proteins were then evaluated in terms of two qualities: *N*-glycosylation and protein cleavage. Major differences were observed between the IFN-γ produced by the baculovirus/silkworm expression system and that produced from the transgenic silkworm expression system. This study introduces new concerns that must be considered when producing biopharmaceutical proteins using *B. mori* as an “insect factory”.

## Results

### Comparison of recombinant IFN-γ production in two protein-expression systems

**Figure 1:**
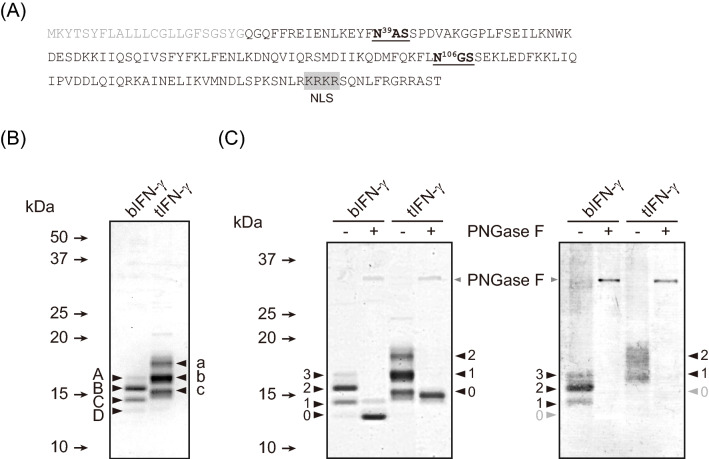
Expression and *N*-glycosylation analysis of IFN-γ. (**A**) Amino acid sequence of the IFN-γs expressed in the baculovirus/silkworm expression system and transgenic silkworm expression system. Gray letters represent the original signal peptide sequence. *N*-Glycosylation sites are shown in bold with underlining. The nuclear localization signal is shaded in gray. The source data for the figure is provided in Supplementary Data File 1. (**B**) CBB staining of purified IFN-γs. Variants of IFN-γ are labeled **A**–**D** in bIFN-γ and a-c in bIFN-γ, respectively. (**C**) De-glycosylation analysis of IFN-γs. The left and right panels represent the results of CBB staining and *N*-glycan staining, respectively. The numbers on both sides represent the electrophoretic migration and the position depending on the *N*-glycosylation state of IFN-γs. PNGase F used in the analysis is shown with gray triangles.

Two major protein-expression systems utilizing *B. mori*, a baculovirus-based expression system using the silkworm as an expression host (baculovirus/silkworm expression system) and a transgenic gene expression system employing microinjection of a gene of interest into eggs (transgenic silkworm expression system), used to express bovine IFN-γ as a model protein. IFN-γ is the primary macrophage-activating cytokine and transduces signals essential for an innate immune response^24^. IFN-γ consists of 166 aa containing a 23 aa N-terminal signal peptide for secretion from activated T lymphocyte cells and also the KRKR sequence for nuclear localization in the region proximal to the C-terminus^25^ (Fig. 1A). IFN-γ possesses two potential *N*-glycosylation sites, Asn39 and Asn106, suggesting that IFN-γ is produced as an *N*-glycoprotein in *B. mori*. It should be noted that *O*-glycosylation of IFN-γ has not yet been identified.

IFN-γ was successfully produced in both systems and purified using cation exchange chromatography. The yields of purified IFN-γ produced by the baculovirus/silkworm expression system and the transgenic silkworm expression system were 580 μg and 2.5 μg per larva, respectively. Thus, focusing only on the production, the baculovirus/silkworm expression system exhibited the superior production system by far. Interestingly, both baculovirus/silkworm expression and transgenic silkworm expression systems produced several different forms of IFN-γ; the transgenic silkworm expression system produced three forms of IFN-γ (tIFN-γ) (bands a to c in Fig. 1B), whereas the baculovirus/silkworm expression system produced four forms of IFN-γ (bIFN-γ) (bands A to D in Fig. 1B and Supplementary Data File 1). IFN-γ has two *N*-glycosylation sites, meaning that three isoforms could be produced in theory: IFN-γ with *N*-glycosylation at both *N*-glycosylation sites, with *N*-glycosylation at either *N*-glycosylation site, and without *N*-glycosylation. To confirm the *N*-glycosylations on IFN-γs, de-glycosylation analysis using peptide: *N*-glycosidase F (PNGase F) and glycan staining were performed (Fig. 1C and Supplementary Data File 1). PNGase F digestion converged *N*-glycosylated tIFN-γs to completely deglycosylated forms which were negative for *N*-glycan staining (Fig. 1C, right panel), whereas bIFN-g still had PNGase F-insesitive *N*-glycans (Fig. 1C, left panel). The molecular mass of the de-glycosylated form of tIFN-γ agreed well with the calculated mass of mature IFN-γ without a signal peptide (16.8 kDa). Meanwhile, deglycosylated bIFN-γ still exhibited two bands, one of which showed the same molecular mass as the de-glycosylated forms of tIFN-γ. In addition, the predominant de-glycosylated form of bIFN-γ was the band showing a faster electro-mobility shift, and the upper de-glycosylated form gave only a faint signal. These results indicated that the transgenic silkworm expression system produced isoforms of IFN-γ with different *N*-glycosylation profiles. However, based on the amino acid sequence of IFN-γ, the four forms detected in bIFN-γ were not caused by *N*-glycosylation itself, because IFN-γ had no other rare potential *N*-glycosylation sites, N-X-C, N-X-V, and N-G^26^ and *C*-mannosylation site, W-X-X-W/C (X represents any amino acid)^27^. This evidence strongly suggested that bIFN-γ was not only *N*-glycosylated but further post-translationally modified by presumably proteolytic degradation(s).

### Structural differences of *N*-Glycan on recombinant IFN-γ

**Figure 2:**
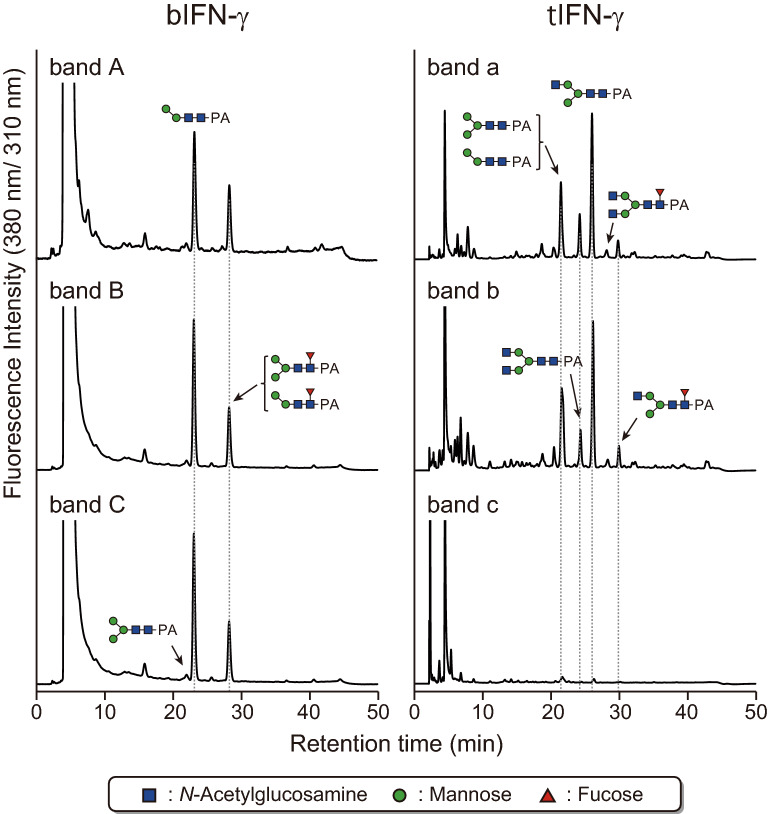
*N*-Glycan analysis of the individual bands in bIFN-γ and tIFN-γ. *N*-Glycans were prepared from each band and labeled with PA. The PA-derivatives were analyzed by RP-HPLC. The major PA-*N*-glycan structures are shown in chromatographs.

Purified IFN-γs from both systems showed not only different molecular masses and numbers of bands but also bands with different features: bIFN-γ showed clear bands, whereas tIFN-γ showed broader bands (Fig. 1B). This suggested two possibilities: either the band differences were attributable to the differences in the number of *N*-glycan structures or to the differences in the structures themselves. That is, either the number of *N*-glycan structures on bIFN-γ was smaller, or the size of the *N*-glycan structures on tIFN-γ was larger, and/or the difference of *N*-glycan structures might have affected the band patterns. In fact, focusing on the *N*-glycan difference, there was a difference of approximately 1 kDa in the molecular masses of the pauci-mannose-type structure(s) and high-mannose-type structures, especially between Man_2_GlcNAc_2_ (Man, mannose; GlcNAc, *N*-acetylglucosamine) (M2) and M8/M9. To investigate the *N*-glycan effect(s) on the variance of IFN-γ, *N*-glycans from each band were prepared and labeled with a fluorescence tag of 2-aminoprydine (PA), then analyzed by reverse phase (RP)-high performance liquid chromatography (HPLC) and liquid chromatography-tandem mass spectrometry (LC–MS/MS) (Fig. 2 and Table 1). Focusing on each IFN-γ, the *N*-glycan structures on each band and their ratio were similar to each other, but they were dissimilar between bIFN-γ and tIFN-γ. Most *N*-glycans on bIFN-γ were pauci-mannose-type structures, whereas the *N*-glycans on tIFN-γ were modified with terminal GlcNAc residue(s). The highest molecular mass *N*-glycan structure was M5 on tIFN-γ, and the ratio was quite low, indicating that the presence of variants detected as different bands was derived not from the difference of *N*-glycans but rather from *N*-glycosylation and/or other post-translational modification(s) of IFN-γs. The predominant *N*-glycans on bIFN-γ and tIFN-γ were M2B and GNM3B, respectively. Notably, the *N*-glycans from bIFN-γ included no GlcNAc-terminal *N*-glycans. The total number of structures on bIFN-γ was much smaller than that of tIFN-γ. These results were also supported by the fact that both the values of *N*-glycan dispersity on bIFN-γ calculated from the molecular weight and their ratio of *N*-glycans were smaller than those for tIFN-γ (Table 1). Thus, *N*-glycan on bIFN-γ had less heterogenicity than that on tIFN-γ.

**Table 1:** Relative amounts of *N*-glycans detected in INF-γs.

Substrate, structure		Abbreviations	Ratio (%)				
			bIFN-γ			tIFN-γ	
			band A	band B	band C	band a	band b
Mannosidic stracture		M2A	1.6	4.7	0.2	–	–
		M2B	48.7	62.0	44.3	5.6	15.2
		M3	3.3	1.3	0.6	22.0	17.7
		M5	–	–	–	1.5	1.9
GlcNAc-terminal structure		GNM2	–	–	–	1.2	1.3
		GNM3A	–	–	–	1.7	0.9
		GNM3B	–	–	–	40.9	35.7
		GN2M3	–	–	–	12.3	9.4
α1,3-Fucosylated structure		MFA	0.9	–	–	–	–
		M2FA	1.2	3.1	5.6	–	–
		GNM3FA	–	–	–	1.7	3.4
α1,6-Fucosylated structure		M2FB	42.8	28.6	48.6	1.6	3.7
		M3FB	1.5	0.3	0.7	3.7	3.6
		GNM3FB	–	–	–	5.8	5.9
		GN2M3FB	–	–	–	2.1	2.1

The relative ratio of the structures was calculated on the basis of the peak area as determined by LC–MS/MS analysis.

### Site-specific *N*-glycosylation analysis of IFN-γ

**Figure 3:**
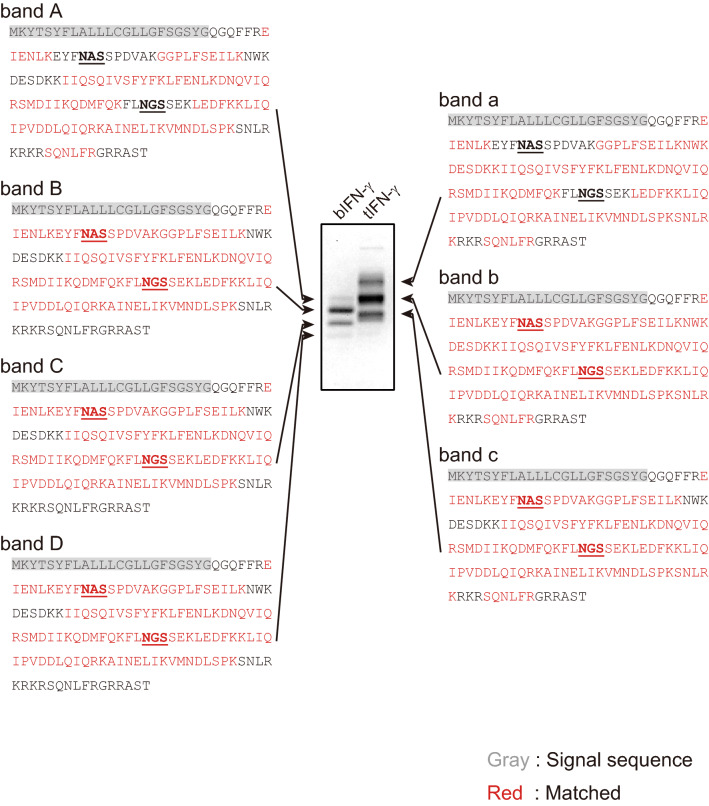
Sequence coverage map of trypsin-digested bIFN-γ and tIFN-γ. The CBB-stained band was excised, digested with trypsin, analyzed by nanoLC-MS/MS and mapped to the expressed sequence of IFN-γ. Mapped sequences were shown in both sides of CBB-stained IFN-γs in Fig. 1B. Letters shown in red, black, and shaded gray are hit and not hit sequences in a MASCOT search, and signal peptide, respectively. The potential *N*-glycosylation sites are shown in bold and underlined.

CBB staining demonstrated that bIFN-γ and tIFN-γ had different *N*-glycosylation variants (Fig. 1b). To confirm the *N*-glycosylation status on Asn39 and Asn106, the bands were further analyzed by tryptic in-gel digestion, followed by nanoLC-MS/MS analysis and MASCOT search (Fig. 3). No signal peptides were detected on bIFN-γ and tIFN-γ, indicating that bIFN-γ and tIFN-γ were secreted in *B. mori* as in mammals. The N-terminal peptide detected in a database search revealed that bIFN-γ and tIFN-γ were modified with the same machinery. Focusing on the bands A and a, neither of the peptides carrying putative *N*-glycosylation sites was hit in the MASCOT search, indicating that Asn39 and Asn106 on bIFN-γ and tIFN-γ were fully *N*-glycosylated. This is because peptide hits in MASCOT search indicates that the MS/MS results are consistent with the calculated molecular weight of the peptide without *N*-glycans. On the other hand, bands B, C, and b were hit in the MASCOT search. De-glycosylation analysis exhibited that these bands were certainly *N*-glycosylated (Fig. 1C), suggesting that the difference of molecular mass of these bIFN-γ and tIFN-γ variants was derived from heterogenicities of *N*-glycosylation; one of the two potential *N*-glycosylation sites was *N*-glycosylated.

**Figure 4:**
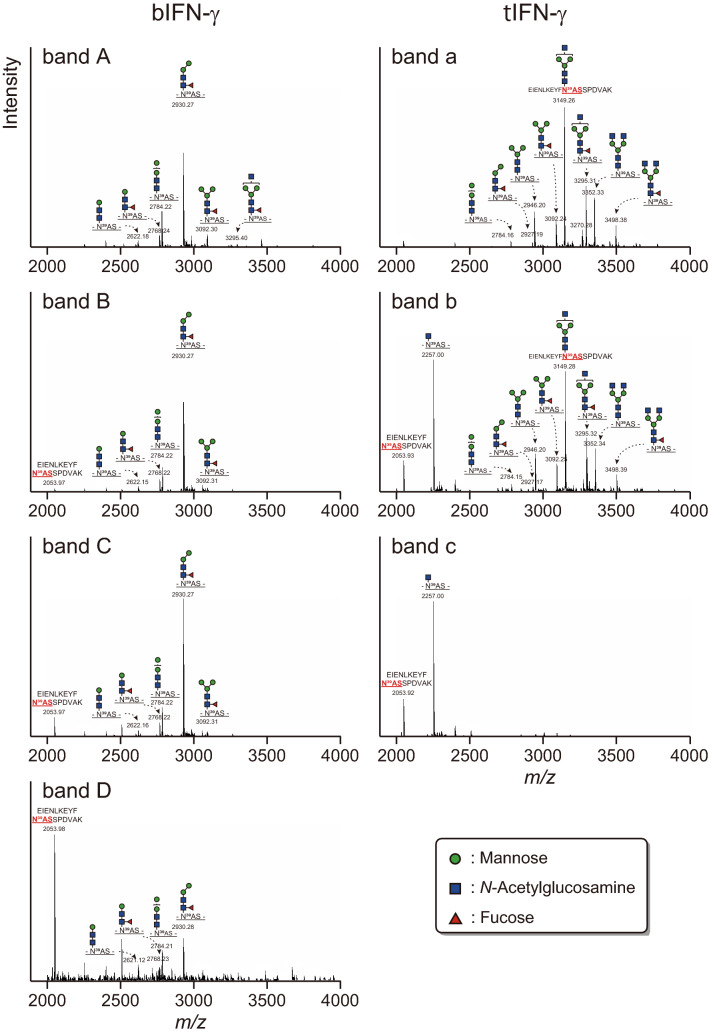
*N*-Glycopeptide analysis of Asn39 in bIFN-γ and tIFN-γ. All signals of *m/z* corresponding to *N*-glycopeptide and the *N*-glycan structures detected in nanoLC-MS/MS analysis are shown.

To elucidate *N*-glycosylation on each peptide in more detail, *N*-glycopeptides were analyzed using datasets from nanoLC-MS/MS analysis (Fig. 4, and Supplemental Fig. S1 and Table S1). Asn39 on bIFN-γ in bands A-C had the M2F structure as the predominant structure, whereas, although some of the *N*-glycan contaminants, possibly from band C, were detected, Asn39 on bIFN-γ in band D was hardly *N*-glycosylated. Through bands A-C, the *N*-glycan structures and their ratio were similar. Focusing on non-*N*-glycosylated Asn39, bands B and C also had the peptide without *N*-glycosylation, *m/z* 2053 from EIENLKEYFN^39^ASSPDVAK, whose ratio increased from band B to D. In Asn106, the predominant structures on each band were M2. Although *N*-glycan structures and their ratio were almost identical to those detected in Asn39, the ratio of non-*N*-glycosylated peptide drastically increased from band B to C, and finally only EIENLKEYFN^39^ASSPDVAK peptide was detected in band D. These results indicated that the *N*-glycosylation variants of band C in bIFN-γ were mainly derived from *N*-glycosylation on Asn39 and non-*N*-glycosylation on Asn106.

In tIFN-γ, band a was fully *N*-glycosylated, and the predominant structure was GNM3 at both Asn39 and Asn106 (Fig. 4 right panel, and Supplemental Fig. S1 and Table S1). However, the *N*-glycan structures and the ratios were slightly different. Asn39 was modified with GlcNAc-terminal and/or fucosylated *N*-glycans, whereas the ratio of GlcNAc-terminal *N*-glycans was decreased by 68% in comparison with Asn39, and fucosylated *N*-glycans were not detected on Asn106. This result demonstrated that the fucosylated *N*-glycans, which were mostly α1,6-linked fucose (Fuc), detected in HPLC analysis in Fig. 2 were derived from Asn39. Interestingly, not only non-*N*-glycosylated peptide but also the peptide retaining a single GlcNAc residue were detected on both *N*-glycosylation sites in band b. Finally, only the non-*N*-glycosylated and predominant peptides with a single GlcNAc residue were detected in band c. The ratios of non-*N*-glycosylated peptides were not significantly changed, but the ratio of the peptides retaining a single GlcNAc residue increased, indicating that the degrees of *N*-glycosylation on Asn39 and Asn106 were approximately similar between bands b and c, but de-glycosylation resulted in the formation of band c from band b. Thus, the decisive difference between band a–c was derived from a further truncation of *N*-glycans on both *N*-glycosylation sites rather than a difference in the *N*-glycosylation state. These results also provided the evidence that most potential *N*-glycosylation sites on tIFN-γ were completely *N*-glycosylated once. It should be noted that this *N*-glycan truncation was not observed in band B–D in bIFN-γ.

### Identification of cleavage sites in IFN-γ

De-glycosylation analysis of IFN-γs by PNGase F digestion revealed two forms of bIFN-γ, suggesting that bIFN-γ had two different peptide backbones (Fig. 1C). The upper band of de-glycosylated bIFN-γ exhibited the same electro-mobility shifts as de-glycosylated tIFN-γ. Peptide mapping analysis of trypsin-digested IFN-γs provided the evidence that the N-terminal peptides were the same among bands A-D (Fig. 3). Remarkably, although the N-terminal peptides detected by peptide mapping were the same, the C-terminal peptide identified in bands a-c, S^155^QNLFR^160^, was identified in band A but not in the peptides from bands B-D. This demonstrated that bIFN-γ consisted of two peptide forms: a mature and full length IFN-γ and a C-terminal-truncated IFN-γ. Furthermore, the major de-glycosylated form of bIFN-γ showed the same electro-mobility shift as band D, indicating that the upper band of de-glycosylated bIFN-γ was from band A and the lower band of de-glycosylated bIFN-γ was from bands B-D. The molecular masses of bands A and a were different, but the estimated difference of approximately 0.92 kDa almost agreed with the difference of the calculated mass of *N*-glycan structures between bIFN-γ and tIFN-γ: 0.73 kDa on average. These results indicated that the peptide backbones of band A in bIFN-γ and tIFN-γ were the same, and the difference in molecular mass between bands A and a was due to the difference of the *N*-glycan structure. Unfortunately, the cleavage site(s) of the C-terminus in bIFN-γ was not determined in this analysis due to the lack of appropriate peptidases for in-gel digestion, but it could be concluded that most of the bIFN-γs were proteolytically-cleaved somewhere in S^147^NLRKRKRSQNLFR^160^.

**Figure 5:**
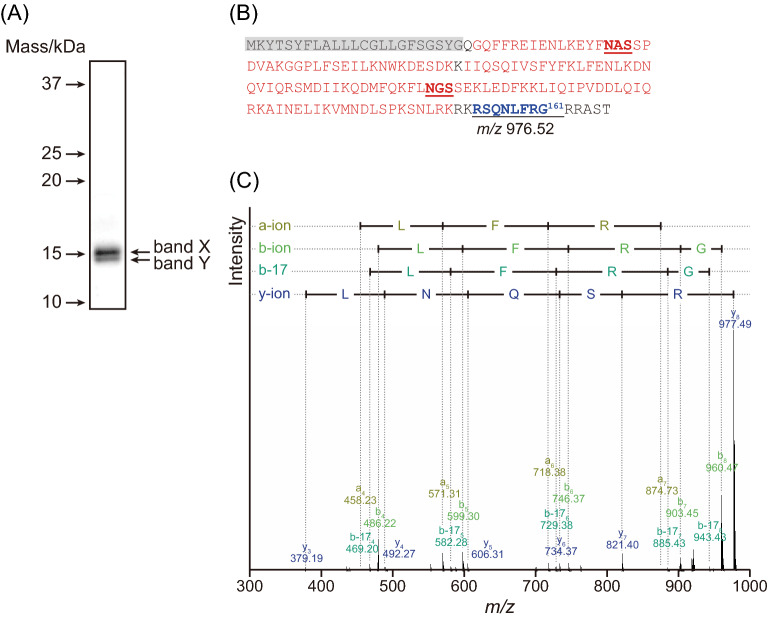
C-terminal analysis of tIFN-γ. (**A**) CBB staining of PNGase F-digested tIFN-γ. The two bands, band X and Y, were excised and digested by Lys-C. The Lys-C-digested product was applied to nanoLC-MS/MS analysis. The source data for the figure is provided in Supplementary Data File 2. (**B**) Sequence coverage map of Lys-C-digested tIFN-γ shown as band Y. Red, black, shaded in gray, and bold and underlined letters are as described in Fig. 3. The calculated *m/z* of Arg154-Gly161, 976.52, is shown. (**C**) MS/MS analysis of the precursor of *m/z* 977.49. a-, b-, b-17, and y-type ions are assigned to the signals detected in MS/MS analysis of the precursor of *m/z* 977.49.

The de-glycosylation product of tIFN-γ also showed two close bands, namely band X and band Y with a ratio of 0.65:0.35 (Fig. 5A). To confirm the cleavage site, both bands were excised, followed by in-gel digestion by Lys-C, nanoLC-MS/MS analysis, and an annotation by peptide mapping (Fig. 5B). Lys-C revealed that the N-terminal sequence of both bands was the same, G^25^QFFREIENLK^35^, suggesting that the C-terminal region of tIFN-γ was cleaved in two different positions. A more detailed search for MS analysis in band Y led to the identification of a peptide corresponding to the mass of SQNL(/I)FRG, *m/z* 976.5. The fragmentation of the peptide by MS/MS demonstrated that the peptide certainly consisted of SQNL(/I)FRG (Fig. 5C). This peptide hit the sequence from Ser154 to Gly161 of IFN-γ. Focusing on the band X, a longer peptide corresponding to the mass of SQNL(/I)FRGRRA, *m/z* 1359.7, was identified and its sequence was also identical (Supplementary Fig. S2). These results indicated that tIFN-γ was cleaved at two different C-terminal sites, Gly161 and Ala164.

## Discussion

Following the first report of IFN-β expression in Sf21 cells using baculovirus in the early 1980s^28^, insects have been widely used as “factories” to produce recombinant proteins^6^. Starting with the first recombinant protein of IFN-α produced using *B. mori* nucleopolyhedrovirus in 1984^13,14^ and the establishment of methods for the generation of transgenic silkworms in 2000^15^, *B. mori* also became a candidate for a promising platform for recombinant protein production. Over the last three decades, therefore, *B. mori* has been used for the production of various kinds of recombinant proteins as model proteins, including fluorescent proteins, immunoglobulins, growth factors and cytokines essential for mammalian proliferation^16,18,19^. In addition, the *B. mori* baculovirus/silkworm expression system produces a target protein and/or peptide within a few days, enabling quick responses to seasonal diseases and epidemics. In fact, the SARS-CoV-2 spike protein has already been produced for research of and/or application to COVID-19 vaccines^29^. Currently, many companies are actively trying to produce recombinant proteins in larvae using baculovirus/silkworm expression systems^30^. Among the commercially available proteins produced in *B. mori* larvae are the IFNs produced by Toray Industries (Tokyo) for veterinary use^30^. In addition to the various efforts to realize rapid and large-scale recombinant protein production by insect baculovirus/silkworm expression systems, *N*-glycosylation of recombinant proteins has also been taken into consideration. *N*-Glycosylation of proteins determines in vivo activities and stabilities, and thus it is imperative insect-type *N*-glycans should be optimized to mammalian type. In the transgenic silkworm expression system, it has become possible to produce a protein-of-interest in each organ using an organ-specific promoter, such as the promoters of *ser1* and *fibH*, thereby controlling the *N*-glycan structure to some extent^31,32^. However, to our knowledge there has been no comprehensive analysis to compare the qualities and quantities of identical proteins expressed in the baculovirus/silkworm expression system and transgenic silkworm expression system. To facilitate the use of the recombinant proteins produced in these two *B. mori* expression systems, more detailed analyses, especially with respect to the protein qualities, will be needed, along with a thorough reevaluation of these two expression systems.

**Figure 6:**
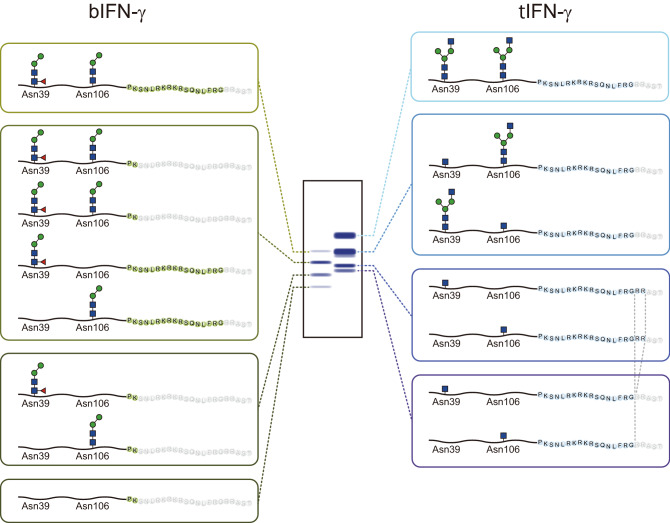
Schematic representation of structural and *N*-glycosylation in IFN-γ variants. Monomeric structures of IFN-γ with *N*-glycans are shown. *N*-Glycans are selected on the basis of the predominant *N*-glycan structure detected in nanoLC-MS/MS analysis. Protein bands are illustrated in accordance with the results of CBB staining in Fig. 1. Sugar residues on *N*-glycan shown in blue, green, and red are GlcNAc, mannose, and α1,6-Fuc, respectively.

Here, focusing on the characteristics of the recombinant proteins, we expressed IFN-γ using two different expression methods in *B. mori*. Both expression systems successfully produced IFN-γ, but the number of variants and their molecular weights were dissimilar. The putative structures of the IFN-γs with *N*-glycans expressed in this study are shown in Fig. 6. A previous study also demonstrated that an insect-produced IFN-γ analyzed using a baculovirus expression vector system showed several bands representing the different levels of *N*-glycosylation^33^. This heterogeneous *N*-glycosylation was due to underglycosylation, and it has also been observed when IFN-γ was expressed in leukocytes^34^, CHO cells^35–38^, and plants^39^. Detailed *N*-glycosylation and *N*-glycan analyses of both bIFN-γ and tIFN-γ revealed that potential *N*-glycosylation sites were *N*-glycosylated in the same manner, but the structures were different; bIFN-γ had pauci-mannose type structures of M2B or M2FB and the number of structures was small, whereas tIFN-γ had GlcNAc-extended structures (Table 1). This was one of the reasons that variants with different molecular weights were produced. The results of *N*-glycan staining also suggested that the number of *N*-glycan structures was smaller on bIFN-γ, since the bands for bIFN-γ were narrower than those for tIFN-γ (Fig. 1C). A previous report demonstrated that INF-γ expressed in Sf9 cells possessed M3 and M3F as predominant structures on Asn39 and Asn 106, respectively^36^. *N*-Glycans on IFN-ω produced in insect cells, as another example, contained M2F and M3F structures, while natural IFN-ω carried complex-type *N*-glycans^40^. Thus, this pauci-mannosidic structure on recombinant *N*-glycoprotein is due to the baculovirus/silkworm expression system. *N*-Glycan of native human IFN-γ produced in CD8^+^ T lymphocytes showed more than 30 *N*-glycan structures, among which some of the *N*-glycan was sialylated with *N*-acetylneuraminic acid^34^. Thus, it is considered to be essential to produce sialylated IFN-γ for in vivo activity. Indeed, IFN-γ was also produced in *E. coli*, but the half-life of *E. coli*-produced IFN-γ was short, presumably due to the underglycosylation caused by lack of *N*-glycosylation^41^. Recombinant IFN-γ with oligo-mannosidic *N*-glycan produced in insect cells had a negative effect on the stability of the IFN-γ in bloodstream circulation, and the IFN-γ was eliminated more rapidly than native IFN-γ^42^. From the point of view of *N*-glycosylation and *N*-glycan, target protein production by a transgenic silkworm expression system enabling organ-specific protein production is preferable. The transgenic silkworm expression system especially in middle silk gland (MSG) resulted in the accumulation of *N*-glycans with terminal GlcNAc residue(s), which is indispensable for biosynthesis of mammalian-type *N*-glycan^31^. Indeed, tIFN-γ expressed in MSG using the GAL4/UAS system and *ser1* promoter had larger amount of *N*-glycans with terminal GlcNAc residues (Table 1 and Supplementary Table S1). Taking into consideration the further *N*-glycosylation and the structural modification of *N*-glycan, it could be concluded that a transgenic silkworm expression system which can produce a protein with an *N*-glycan structure closer to that of the mammalian type is more suitable for protein production compared to a baculovirus/silkworm expression system with a high production level.

Interestingly, a single GlcNAc residue on an *N*-glycosylation site was detected in both Asn39 and Asn106 of tIFN-γ. This unusual GlcNAc modification has also been reported in some plant proteins^43,44^. As mentioned above, heterologously expressed IFN-γs had *N*-glycosylation variants due to the underglycosylation^34,35,37–39^, but this single GlcNAc residue on *N*-glycosylation sites and the presence of IFN-γ with different molecular masses due to this single GlcNAc modification has not been reported so far. In general, sequential reactions of glycosyltransferases of ALG family proteins in the ER catalyze the biosynthesis of Glc_3_Man_9_GlcNAc_2_ on dolichol (Dol)^45^ and the intermediates of this oligosaccharide are hardly detected except for the *alg* mutant. Glc_3_Man_9_GlcNAc_2_-PP-Dol, the most optimal substrate of the oligosaccharyltransferase (OST), is transferred to nascent polypeptides by the action of OST complex. In this oligosaccharide transfer, the terminal α1,2-linked glucose is necessary for efficient *N*-glycosylation mediated by OST^46^. GlcNAc_2_-PP-Dol is also used as a minimal donor substrate, however, GlcNAc-PP-Dol is poorly utilized by OST compared to GlcNAc_2_-PP-Dol although this GlcNAc residue is important for the catalytic function of OST^47^. These facts suggest that the GlcNAc residue on tIFN-γ is not synthesized by incomplete *N*-glycosylation using GlcNAc-PP-Dol as a donor, but is a product that is decomposed after sugar chain synthesis. Therefore, *B. mori* might have endoglycosidase(s) contributing to the hydrolysis of the glycosidic bond between R-GlcNAcβ1,4-GlcNAc-Asn. One candidate for the endoglycosidase is *endo*-β-*N*-acetylglucosaminidase (ENGase). ENGase is categorized into two glycoside hydrolase (GH) families, GH18 and GH85, in the carbohydrate-active enzymes (CAZy) database (http://www.cazy.org). GH85 is widely distributed from bacteria, through fungi, to eucaryotes^48^. Insects also have ENGase, and a putative *B. mori* ENGase (Gene Model ID KWMTBOMO10033) was also identified as a GH85 protein in a database search using KAIKObase (https://kaikobase.dna.affrc.go.jp/). Thus, *N*-glycans on tIFN-γ might be cleaved off by the action of this putative *B. mori* ENGase. It has hypothesized that this ENGase contribution to the production of a single GlcNAc modification also occurs in plants^43,44^. This uncommon modification of the GlcNAc residue on *N*-glycosylation site(s) would also affect the stability of tIFN-γ. However, the GlcNAc residue also could be applied to *N*-glycoprotein remodeling by chemoenzymatic transglycosylation. Transglycosylation mediated by ENGase activities with highly active intermediates, *e.g.*, glycan oxazolines, has been applied to the chemoenzymatic synthesis of *N*-glycoproteins with a structure consisting of homogeneous *N*-glycoforms^49,50^. Therefore, modification of the GlcNAc residue on tIFN-γ with sialylated *N*-glycans by transglycosylation would enable the production of more reliable IFN-γ with improved stability in bloodstream circulation.

The most significant and critical difference in the IFN-γs produced by the two different systems concerned the cleavage and/or degradation of the C-terminal region (Fig. 5). IFN-γ has two important peptides: a secretion signal at the N-terminus and a propeptide at the C-terminus^41^. The C-terminal sequence of tIFN-γ, S^154^QNLFRG^161^, agreed well with the native IFN-γ, suggesting that *B. mori* has the same IFN-γ maturation machinery as mammals. Therefore, from the protein-maturation point of view, the transgenic silkworm expression system using genetic transformation is preferable to the baculovirus/silkworm expression system. On the other hand, S^154^QNLFRGRRA^164^ was also detected, suggesting tIFN-γ might be maturated via two different machineries in *B. mori*, although if so, the details are not yet clear. This was also suggested by the results for bIFN-γ, because bIFN-γ was produced as a more truncated, proteolytically-cleaved or degraded form than tIFN-γ. The C-terminal effect of INFγ on the activity has been controversial. In fact, some C-terminal-truncated variants, which are presumably the same as those observed in tIFNγ, have been reported in natural IFN-γ^51,52^, and this unexpected C-terminal nine-amino-acid truncation was also observed in insect-produced IFN-ω^40^. However, a previous report demonstrated that truncation of the C-terminal flexible domain consisting mainly of Lys and Arg in IFN-γ contributes to the stability of the molecule, and thereby enhances the solubility of IFN-γ^53^, but this truncation of the C-terminal flexible domain also induces a substantial abolishment of biological activity, a phenomenon which becomes significant when there are twelve or more amino-acid deletions from the C-terminus^53^. Furthermore, bIFN-γ presumably lacked K^150^RKR^153^, which functions as a nuclear localization signal and is essential for the nuclear translocation and cytokine function of IFN-γ^25,54^. These facts suggested that bIFN-γ is less functional and/or has no in vivo activity due to the loss of the C-terminus. It has not been clarified whether this C-terminal truncation occurs only in IFN or also takes place in other proteins when the target protein is expressed using a baculovirus expression system. As for the truncation of several amino acid residues, the higher the molecular mass of the produced protein, the more difficult it is to evaluate the difference from that of the native protein, which results in several amino acid truncations being overlooked. Although this phenomenon might be limited to IFN-γ, and other model proteins expressed in *B. mori* should be evaluated, the truncation of amino acids in the baculovirus expression system might need to be taken into consideration, along with post-translational modifications in protein production.

## Methods

### Materials

The YMC*GEL Silica 12 nm S-50 μm column was purchased from YMC (Kyoto, Japan). Chelating Sepharose FF, the HiPrep 26/10 desalting column (Cytiva, Tokyo, Japan), HiPrep 16/10 S FF, and HiLoad 16/10 SP Sepharose High Performance were purchased from Cytiva.

2-Aminoprydine (PA) was purchased from FUJIFILM Wako Chemicals (Osaka, Japan). PA-labeled sugar chains were purchased from TaKaRa Bio (Shiga, Japan) and Masuda Chemical Industry (Kagawa, Japan).

### Expression, purification, and quantification of IFN-γ

For the production and purification of bIFN-γ, the recombinant virus of cathepsin-deficient strain was injected into 50 silkworm larvae at the second day of fifth instar (5.0 × 10^4^ pfu/larva). Six days after injection, hemolymph was collected with the addition of a small amount of phenylthiourea (FUJIFILM Wako). Then, the harvested hemolymph was clarified by low-speed centrifugation and subsequent ultracentrifugation. The supernatant of hemolymph was diluted 1:4 with 50 mM Tris–HCl pH 8.0 and applied on the YMC*GEL Silica 12 nm S-50 μm column, which was equilibrated with 50 mM Tris–HCl pH 8.0. After washing the column with 50 mM Tris–HCl pH 8.0 containing 1 M NaCl, bIFN-γ was eluted with 50 mM Tris–HCl pH 8.0 containing 3 M NaCl and 30% ethylene glycol. The bIFN-γ fractions were diluted 1:9 with 50 mM phosphate buffer pH 7.0 and applied on the Chelating Sepharose FF column with CuSO_4_-5H_2_O, which was equilibrated with 50 mM phosphate buffer pH7.0. The purified bIFN-γ was recovered with 50 mM phosphate buffer pH7.0 and concentrated with VIVASPIN 20 MWC 10,000 (Cytiva). Concentrated bIFN-γ was desalted to DW with a HiPrep 26/10 desalting column. The amount of protein was quantified by using a BCA Protein Assay kit (Thermo Fisher Scientific, Tokyo, Japan).

For the transgenic silkworm expression, the bovine IFN-γ gene was amplified from the ORFeome Collaboration clone, ORH24802P (Promega, Madison, WI). The *Bln*I fragment of this PCR product was inserted into the *Bln*I site of pBac[SerUAS/3xP3EGFP]^55^ to generate pBac[SerUAS_IFN-γ/3xP3-EYFP]. Transgenic silkworms were generated as previously reported using the plasmid pBac[SerUAS_IFN-γ/3xP3-EYFP] as a vector^15^. Then the obtained transgenic silkworm lines harboring the IFN-γ gene under the regulation of a UAS sequence were mated with adults from the Ser1-GAL4 strain, which carried a GAL4 gene driven by the sericin1 promoter.

The middle glands isolated from transgenic silkworms were immersed in 20 mM phosphate-buffer, pH 7.2, and gently shaken for 90 min in an ice bath. The resulting extract was centrifuged at 10,000×*g* for 30 min and the supernatant fraction containing the extracted proteins was collected. The extraction solution was frozen overnight, and the high-molecular-weight fiber proteins precipitated after thawing were removed by centrifugation. The extracted solution was coarsely fractionated by ammonium sulfate at a concentration of 60%-80%. The precipitated crude fraction components were subjected to intermediate purification by cation chromatography using a cation exchange column (HiPrep 16/10 S FF). Fractions with high expression product content were subjected to final purification using a cation exchange column (HiLoad 16/10 SP Sepharose High Performance).

### *N*-Glycosylation analysis of IFN-γ

*N*-Glycans on IFN-γs were digested using Peptide:*N*-glycosidase F (TaKaRa Bio, Shiga, Japan). *N*-Glycosylated and de-glycosylated IFN-γs were desalted and concentrated by acetone precipitation, followed by SDS-PAGE separation using a SuperSep™ Ace 15% Gel (FUJIFILM Wako Chemicals, Osaka, Japan), CBB R-250 staining or *N*-glycan staining using G.P.Sensor (J-OIL MILLS, Tokyo, Japan) and a POD immunostain kit (FUJIFILM Wako Chemicals). Digital images of CBB staining gels and stained blots were obtained using scanner software and processed via Adobe Photoshop v. 22.5.0. The ratio of the two PNGase F-digested tIFN-γ s was determined from their band intensities using Image J^56^.

### *N*-Glycan analysis of IFN-γ

IFN-γs were separated by SDS-PAGE using a SuperSep™ Ace 15% Gel and stained with CBB R-250. The CBB-stained bands corresponding to the IFN-γs were excised from the gel and cut into small pieces, then de-stained completely with 50 mM NH_4_HCO_3_ in 50% acetonitrile and dehydrated twice using acetonitrile. The proteins in the gels were in-gel digested using Trypsin Gold (Promega) in ProteaseMAX™ Surfactant (Promega) at 50 °C for 1 h. The supernatants and extracts of gel pieces dehydrated using acetonitrile were lyophilized overnight. The resulting material was subjected to hydrazinolysis, followed by lyophilization, *N*-acetylation, desalting with Dowex 50 × 2 (Muromachi Kagaku Kogyo, Kyoto, Japan), PA-labeling, and purification^57^.

The PA-sugar chains were detected by RP-HPLC using a HITACHI LaChrom HPLC system equipped with fluorescence^58^. Briefly, the mobile phase was composed of 0.02% trifluoroacetic acid (solvent A) and acetonitrile/0.02% trifluoroacetic acid (solvent B) (20/80, v/v). RP-HPLC was performed using a Cosmosil 5C_18_-AR-II column (4.6 × 250 mm; Nacalai Tesque, Kyoto, Japan) with a HITACHI LaChrom HPLC system by linearly increasing the solvent B concentration from 0 to 25% over 25 min at a flow rate of 0.7 ml/min. The eluted fractions were monitored by measuring the fluorescence intensity using excitation and emission wavelengths of 310 and 380 nm, respectively.

### LC–MS/MS analysis and structural determination of PA-sugar chains

The structural determination of PA-sugar chains chain was performed as previously reported^58^. Briefly, LC–MS/MS was performed using an Agilent Technologies 1200 series instrument (Agilent Technologies, Santa Clara, CA) equipped with HCT plus software (Bruker Daltonics, Bremen, Germany). For the LC, the mobile phase was composed of acetonitrile/acetic acid (solvent A: 98/2, v/v) and water/acetic acid/triethylamine (solvent B: 92/5/3, v/v/v). A Shodex Asahipak NH2P-50 2D column (2.0 mm ID × 150 mm; SHOWA DENKO) was used as an analytical column. The concentration of solvent B was increased in a linear gradient the concentration from 20 to 55% over 35 min at a flow rate of 0.2 mL/min. The MS/MS analysis was performed in the positive-ion mode using the following parameters: scan range *m/z* 350–2750; nebulizer flow of 5.0 psi; dry gas flow rate of 3.0 L/min; dry temperature of 300 °C; target count of 200,000; and MS/MS Frag. Ampl. of 1.0 V. The relative amount of *N*-glycan was calculated on the basis of the peak area of the LC.

Based on the possible structure deduced from LC–MS/MS analysis, each structure of PA-sugar chains separated and collected using normal phase-HPLC^58^ was compared with that of authentic PA-sugar chains. Non-commercially available PA-sugar chains were prepared as previously reported using glycosidases, such as mannosidase and *N*-acetyl-hexosaminidase, and commercially available or prepared PA-sugar chains from *B. mori* ^58^.

### Peptide and *N*-glycopeptide analysis of IFN-γ

*N*-Glycopeptide analysis was performed in the same manner as previously reported^58^. Briefly, small gel pieces of IFN-γ were prepared as described above. The proteins in the gels were in-gel digested using Trypsin Gold (Promega) in ProteaseMAX™ Surfactant (Promega) at 50 °C for 1 h or Lys-C (Promega) in ProteaseMAX™ Surfactant at 37 °C for 20 h. The supernatant was collected, and the reactions were terminated by the addition of trifluoroacetic acid to a final concentration of 0.5%. The trypsinized or Lys-C digested products were analyzed using an Agilent Technologies 1200 series nanoLC system (Agilent Technologies) equipped with a micrOTOF-QII TOF–MS (Bruker Daltonics). For the liquid chromatography portion of the analysis, ZORBAX 300SB-C18 (5 μm, 0.3 mm × 5 mm) and ZORBAX 300SBC18 (3.5 mm, 75 μm × 150 mm) (Agilent Technologies) columns were used as the trapping and analytical column, respectively. The mobile phase was composed of 0.1% formic acid (solvent A) and acetonitrile containing 0.1% formic acid (solvent B) for nanoLC and 0.1% trifluoroacetic acid (solvent C) for peptide trapping on the column. Following injection, the flow was directed to the trapping column in solvent C at a flow rate of 10 μL/min. The peptides were separated by linearly increasing the solvent B concentration from 8 to 30% over 30 min at a flow rate of 600 nL/min, followed by washing with 95% solvent B for 5 min, and equilibration for 18 min at the initial flow. In the MS portion of the analysis, the MS/MS parameters were as follows: scan range *m/z* 50–4500; nebulizer flow of 1.0 bar; dry gas flow rate of 5.0 L/min; and dry temperature of 180 °C in the positive-ion mode. The MS data were analyzed using Data Analysis 4.0 software, BioTools, and SequenceEditor (Bruker Daltonics).

### *N*-Glycoprotein modeling

The *N*-glycoprotein models of IFN-γs with *N*-glycans were constructed using bovine IFN-γ (PDB id: 1D9C) as a protein template and the GLYCAM server (http://glycam.org/). Figures were prepared using the PyMOL molecular graphics system, version 1.7.1.1. (http://www.pymol.org/).

## Supplementary Information

Supplementary Information 1.

Supplementary Information 2.

## Data Availability

The datasets used and/or analyzed during the current study available from the corresponding author on reasonable request.
