# Supplementary material for: Insights into the quality of recombinant proteins produced by two different Bombyx mori expression systems
Source: Sci Rep. 2022 Nov 2;12:18502. doi: 10.1038/s41598-022-22565-7 (PMC9628610; doi:10.1038/s41598-022-22565-7)
Supplement: Supplementary file 1 — Supplementary Information 1. [file 41598_2022_22565_MOESM1_ESM.pdf]

Insights into the quality of recombinant proteins

produced by two different *Bombyx mori* expression systems

Supplementary Data File

Hiroyuki Kajiura[1,2,†], Ken-ichiro Tatematsu[3,†], Tsuyoshi Nomura[4,†], Mitsuhiro Miyazawa[5], Akihiro Usami[4], Toshiki Tamura[6], Hideki Sezutsu[3], Kazuhito Fujiyama[1,2,7]*

[1]International Center for Biotechnology, Osaka University, 2-1 Yamada-oka, Suita-shi, Osaka, 565-0871, Japan

[2]Institute for Open and Transdisciplinary Research Initiatives (OTRI), Osaka University, 2-1 Yamada-oka, Suita-shi, Osaka 565-0871, Japan

[3]Division of Silk-Producing Insect Biotechnology, Institute of Agrobiological Sciences, National Agriculture and Food Research Organization, 1-2 Owashi, Tsukuba, Ibaraki 305-8634, Japan

[4]Sysmex Corporation, 1548 Ooaza Shimookudomi, Sayama, Saitama 350-1332, Japan

[5]Division of Biomaterial Sciences, Institute of Agrobiological Sciences, National Agriculture and Food Research Organization, 1-2 Owashi, Tsukuba, Ibaraki 305-8634, Japan

[6]Silk Science and Technology Research Institute, 1053, Iikura, Ami-machi, Ibaraki, 300-0324, Japan

[7]Osaka University Cooperative Research Station in Southeast Asia (OU:CRS), Faculty of Science, Mahidol University, Bangkok, Thailand

†: These authors equally contributed to this work.

*Correspondence should be addressed to K.F. (fujiyama@icb.osaka-u.ac.jp)

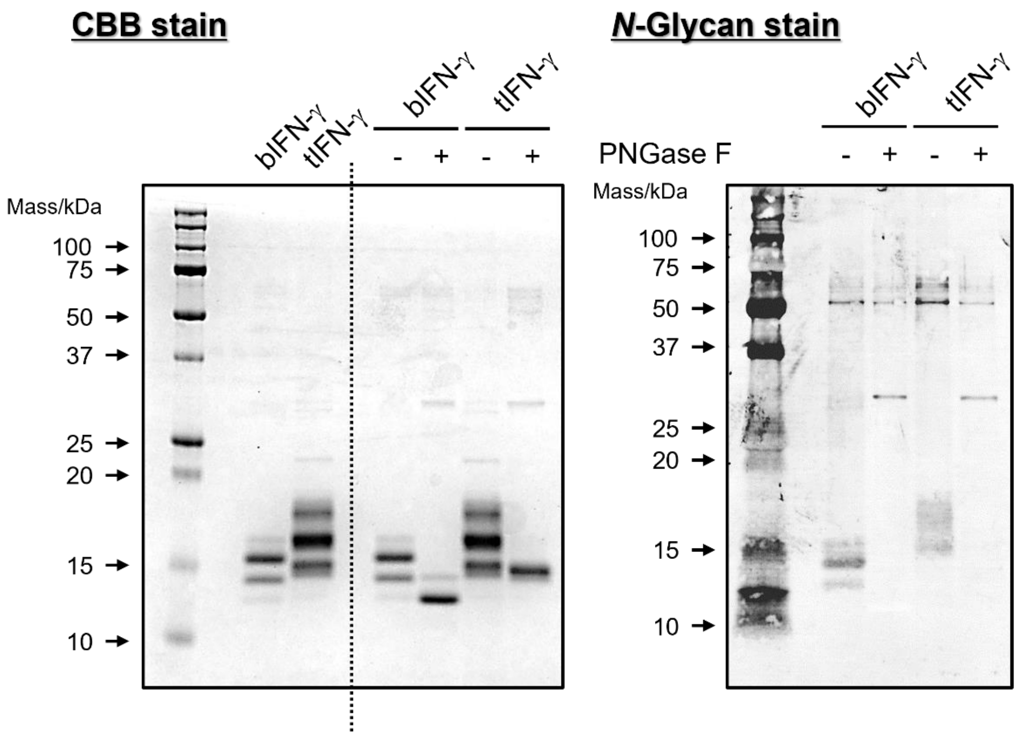

Supplementary Data File 1 Original figures of CBB staining and *N*-glycan staining.

Of the figure divided by the dotted line of CBB staining, the left side was used for Fig. 1b and Fig. 2, and the right side was used for Fig. 1c. *N*-Glycan staining result was cropped to remove unnecessary region and was used for Fig. 1c.

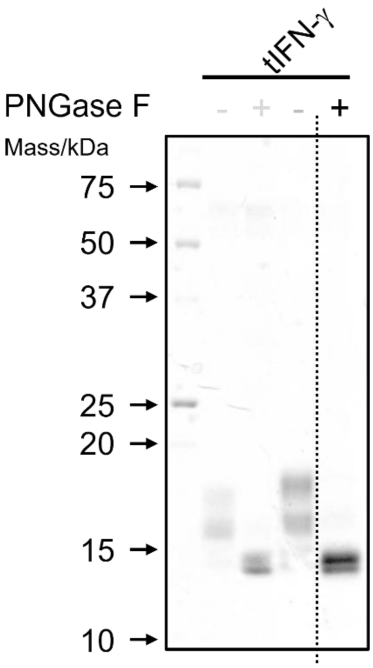

Supplementary Data File 2 Original figure of de-glycosylated tIFN-γ

Two different concentrations of purified tIFN-γ were separated by SDS-PAGE, followed by CBB staining. Of the figure divided by the dotted line, the right side was used for Fig. 5a. The edges of the gel were cut off and not shown because the image was acquired by specifying an image range during the scanning process.
