# Supplementary material for: Insights into the quality of recombinant proteins produced by two different Bombyx mori expression systems
Source: Sci Rep. 2022 Nov 2;12:18502. doi: 10.1038/s41598-022-22565-7 (PMC9628610; doi:10.1038/s41598-022-22565-7)
Supplement: Supplementary file 2 — Supplementary Information 2. [file 41598_2022_22565_MOESM2_ESM.pdf]

Insights into the quality of recombinant proteins

produced by two different *Bombyx mori* expression systems

Supplementary Information

Hiroyuki Kajiura[1,2,†], Ken-ichiro Tatematsu[3,†], Tsuyoshi Nomura[4,†], Mitsuhiro Miyazawa[5], Akihiro Usami[4], Toshiki Tamura[6], Hideki Sezutsu[3], Kazuhito Fujiyama[1,2,7]*

[1]International Center for Biotechnology, Osaka University, 2-1 Yamada-oka, Suita-shi, Osaka, 565-0871, Japan

[2]Institute for Open and Transdisciplinary Research Initiatives (OTRI), Osaka University, 2-1 Yamada-oka, Suita-shi, Osaka 565-0871, Japan

[3]Division of Silk-Producing Insect Biotechnology, Institute of Agrobiological Sciences, National Agriculture and Food Research Organization, 1-2 Owashi, Tsukuba, Ibaraki 305-8634, Japan

[4]Sysmex Corporation, 1548 Ooaza Shimookudomi, Sayama, Saitama 350-1332, Japan

[5]Division of Biomaterial Sciences, Institute of Agrobiological Sciences, National Agriculture and Food Research Organization, 1-2 Owashi, Tsukuba, Ibaraki 305-8634, Japan

[6]Silk Science and Technology Research Institute, 1053, Iikura, Ami-machi, Ibaraki, 300-0324, Japan

[7]Osaka University Cooperative Research Station in Southeast Asia (OU:CRS), Faculty of Science, Mahidol University, Bangkok, Thailand

†: These authors equally contributed to this work.

*Correspondence should be addressed to K.F. (fujiyama@icb.osaka-u.ac.jp)

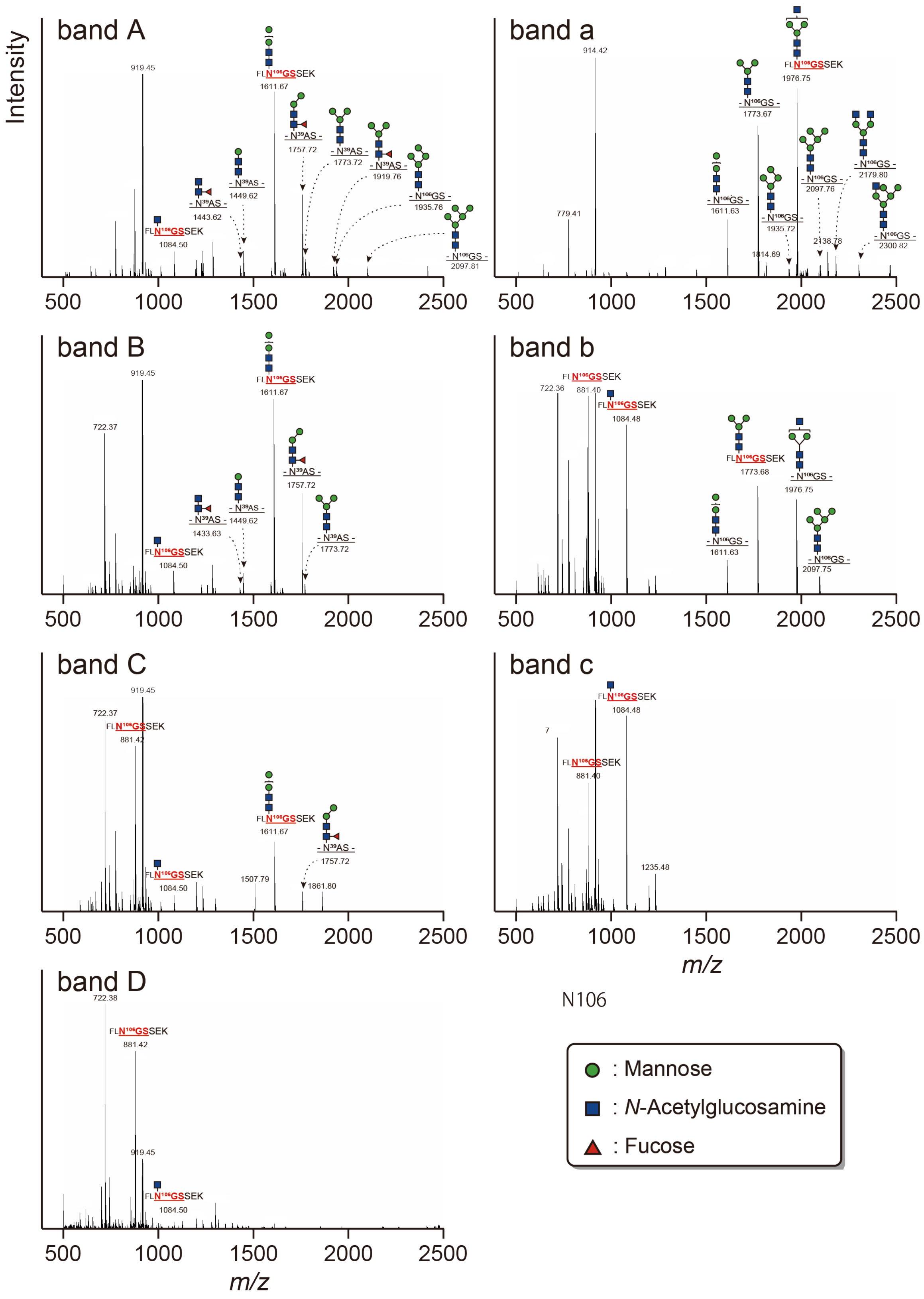

Supplementary Figure S1 ***N*-Glycopeptide analysis of Asn106 in bIFN-γ and tIFN-γ**

All signals of *m/z* corresponding to *N*-glycopeptide and the *N*-glycan structures detected in nanoLC-MS/MS analysis are shown.

(A)

MKYTSYFLALLLCGLLGFSGSYGQGQFFREIENLKEYF**NAS**SP
DVAKGGPLFSEILKNWKDESDKKIIQSQIVSFYFKLFENLKDN
QVIQRSMDIIKQDMFQKFL**NGS**SEKLEDFKKLIQIPVDDLQIQ
RKAINELIKVMNDLSPKSNLRKRK**RSQNLFRGRRA**^{164}ST
m/z 1359.76

(B)

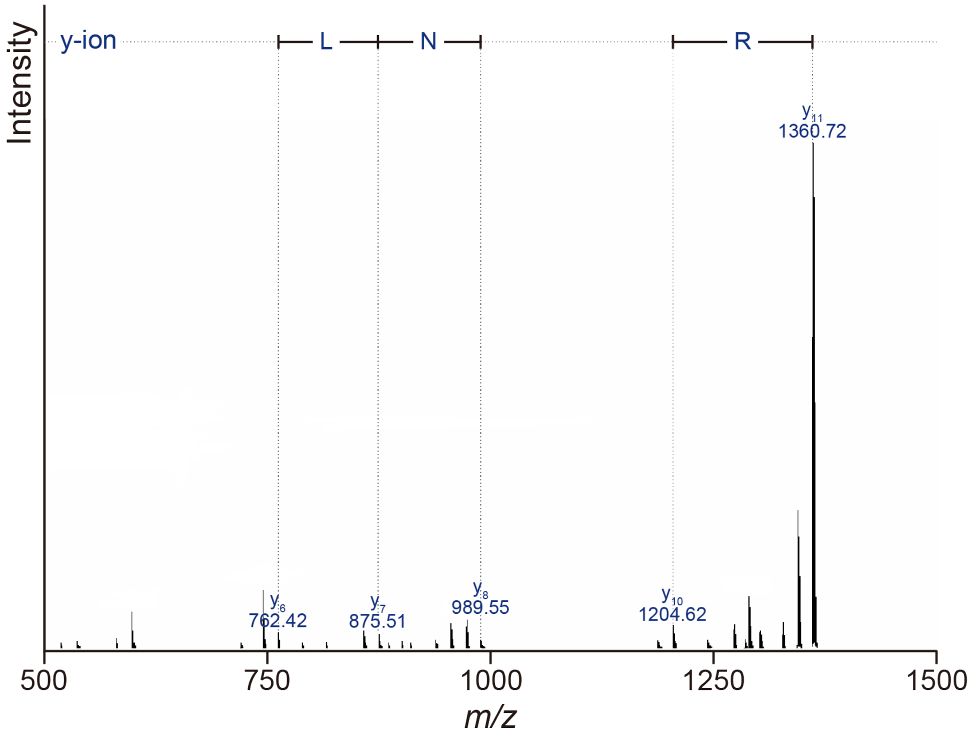

Supplementary Figure S2 C-terminal analysis of tIFN-γ

(a) Sequence coverage map of Lys-C-digested tIFN-γ shown as band X in Fig. 5(a). Red, black, shaded in gray, and bold letters are as described in Fig. 3. The calculated *m/z* of Arg154-Gly164, 1359.76, is shown.

(b) MS/MS analysis of the precursor of *m/z* 1359.72. y-type ions are assigned to the signals detected in MS/MS analysis of the precursor of *m/z* 1359.72.

Supplementary Table S1 Site-specific *N*-glycan structures of bIFN-γ and tIFN-γ and their relative ratios

Structure			Ratio (%)													
		Expression system	bIFN-γ								tIFN-γ					
		Band	A		B		C		D		a		b		c	
		N-Glycosylation site	Asn39	Asn106	Asn39	Asn106	Asn39	Asn106	Asn39	Asn106	Asn39	Asn106	Asn39	Asn106	Asn39	Asn106
Peptide	EIENLKEYF**N**^{39}**AS**SPDVAK		-	-	1.2	-	8.4	-	56.8	-	-	-	6.8	-	3.8	-
	FL**N**^{106}**GS**SEK		-	-	-	4.1	-	60.1	-	96.5	-	-	-	25.8	-	32.2
	$HexNAc_1$		1.7	5.4	0.9	5.5	2.1	5.9	-	3.5	-	-	27.9	29.1	96.2	67.8
	$HexNAc_2$		-	8.1	-	7.0	0.7	-	-	-	-	-	-	-	-	-
	$DeoxyHex_1HexNAc_2$		-	2.9	-	1.5	2.6	-	-	-	-	-	-	-	-	-
Mannose type *N*-glycan	$Hex_1HexNAc_2$	M1	2.5	5.8	2.5	5.1	2.6	-	6.6	-	-	1.8	-	-	-	-
	$Hex_2HexNAc_2$	M2	14.4	41.9	15.2	46.5	12.5	26.4	12.8	-	1.7	11.7	1.5	5.9	-	-
	$Hex_3HexNAc_2$	M3	-	4.2	2.1	2.4	2.6	-	-	-	9.8	31.6	7.7	19.5	-	-
	$Hex_4HexNAc_2$	M4	-	2.4	-	-	-	-	-	-	-	1.4	-	-	-	-
	$Hex_5HexNAc_2$	M5	-	2.1	-	-	-	-	-	-	4.8	2.3	2.4	3.1	-	-
Fucosylated *N*-glycan	$Hex_1DeoxyHex_1HexNAc_2$	MF	5.9	3.0	6.3	2.9	5.8	-	6.8	-	-	-	-	-	-	-
	$Hex_2DeoxyHex_1HexNAc_2$	M2F	68.1	21.9	69.2	25.0	59.5	7.6	17.1	-	1.2	-	-	-	-	-
	$Hex_2DeoxyHex_2HexNAc_2$	M2FF	1.4	-	0.9	-	0.7	-	-	-	7.2	-	5.7	-	-	-
	$Hex_3DeoxyHex_1HexNAc_2$	M3F	6.1	2.5	1.9	-	2.4	-	-	-	0.0	-	-	-	-	-
Terminal GlcNAc *N*-glycan	$Hex_3HexNAc_3$	GNM3	-	-	-	-	-	-	-	-	38.6	40.2	24.8	16.6	-	-
	$Hex_4HexNAc_3$	GNM4	-	-	-	-	-	-	-	-	-	4.6	-	-	-	-
	$Hex_5HexNAc_3$	GNM5	-	-	-	-	-	-	-	-	-	2.4	-	-	-	-
	$Hex_3DeoxyHex_1HexNAc_3$	GNM3F	-	-	-	-	-	-	-	-	16.9	-	10.8	-	-	-
	$Hex_3HexNAc_4$	GN2M3	-	-	-	-	-	-	-	-	13.8	4.0	8.7	-	-	-
	$Hex_3DeoxyHex_1HexNAc_4$	GN2M3F	-	-	-	-	-	-	-	-	6.1	-	3.5	-	-	-
	Total	Mannose-type *N*-glycan	16.8	56.2	19.7	53.9	17.7	26.4	19.3	-	16.2	48.8	11.7	28.5	-	-
		Fucosylated *N*-glycan	81.5	27.4	78.2	27.9	68.5	7.6	23.9	-	31.3	-	20.1	-	-	-
		Terminal GlcNAc *N*-glycan	-	-	-	-	-	-	-	-	75.4	51.2	47.8	16.6	-	-

N-Glycan ratios were calculated on the basis of the intensities of signals.

M: Mannose; GN: *N*-Acetylglucosamine; F: Fucose
